# Cytoplasmic Accumulation and Aggregation of TDP-43 upon Proteasome Inhibition in Cultured Neurons

**DOI:** 10.1371/journal.pone.0022850

**Published:** 2011-07-29

**Authors:** Janet van Eersel, Yazi D. Ke, Amadeus Gladbach, Mian Bi, Jürgen Götz, Jillian J. Kril, Lars M. Ittner

**Affiliations:** 1 Laboratory for Translational Neurodegeneration, Brain & Mind Research Institute, University of Sydney, Sydney, Australia; 2 Alzheimer's and Parkinson's Disease Laboratory, Brain & Mind Research Institute, University of Sydney, Sydney, Australia; 3 Disciplines of Medicine and Pathology, University of Sydney, Sydney, Australia; Thomas Jefferson University, United States of America

## Abstract

Amyotrophic lateral sclerosis (ALS) and frontotemporal lobar degeneration (FTLD) are characterized by intraneuronal deposition of the nuclear TAR DNA-binding protein 43 (TDP-43) caused by unknown mechanisms. Here, we studied TDP-43 in primary neurons under different stress conditions and found that only proteasome inhibition by MG-132 or lactacystin could induce significant cytoplasmic accumulation of TDP-43, a histopathological hallmark in disease. This cytoplasmic accumulation was accompanied by phosphorylation, ubiquitination and aggregation of TDP-43, recapitulating major features of disease. Proteasome inhibition produced similar effects in both hippocampal and cortical neurons, as well as in immortalized motor neurons. To determine the contribution of TDP-43 to cell death, we reduced TDP-43 expression using small interfering RNA (siRNA), and found that reduced levels of TDP-43 dose-dependently rendered neurons more vulnerable to MG-132. Taken together, our data suggests a role for the proteasome in subcellular localization of TDP-43, and possibly in disease.

## Introduction

Amyotrophic lateral sclerosis (ALS) [also referred to as Lou Gehrig's disease] is the most frequent form of motor neuron disease and is characterized by rapidly progressive degeneration of upper and lower motor neurons. ALS often leads to death within three years of diagnosis [1], [2]. Unfortunately, there is no cure available. In comparison, frontotemporal lobar degeneration (FTLD) is a heterogeneous group of neurodegenerative disorders, clinically characterized by behavioral changes and/or language abnormalities, which may also be accompanied by motor symptoms, such as parkinsonism [3], [4] or ALS [3], [5]. FTLD is the second most common form of dementia presenting before the age of 65 [6]. In contrast to Alzheimer's disease, memory deficits are less common in FTLD [7], [8]. As for ALS, no therapy or cure is available for FTLD.

Histopathologically, FTLD can either present with inclusions consisting of fibrillary aggregates of the microtubule-associated protein tau, or inclusions which are tau-negative, but stain with antibodies to ubiquitin (FTLD-U) [9]. The FTLD-U cases can be further categorized based on the protein content of the ubiquitinated inclusions [5]. Hence, FTLD-U cases with inclusions consisting of the protein TAR DNA-binding protein 43 (TDP-43) are termed FTLD-TDP, in contrast to FTLD-tau with tau inclusions, FTLD-FUS with inclusions containing fused in sarcoma (FUS) protein, and a smaller group of cases with deposits of unknown content termed FTLD-ubiquitin proteasome system [10]. Similar ubiquitin-positive inclusions are also found in motor neurons and some other neurons in ALS, and can contain either aggregated TDP-43 [11], [12] and/or FUS [13]. Furthermore, TDP-43 pathology is also observed in a proportion of patients with other neurodegenerative disorders such as Alzheimer's disease, but in a restricted anatomical distribution and at a much lower density [14]. In FTLD-TDP and ALS, the cytoplasmic accumulation of TDP-43 is accompanied by loss of staining of the nucleus, suggesting that loss of its nuclear function may contribute to the pathogenesis of the disease. The mechanisms, however, underlying this redistribution from the nuclear to the cytoplasmic compartment, as well as the cellular consequences, remain to be elucidated.

TDP-43 is an ubiquitous nuclear protein of 414 amino acids and 43 kD that is encoded by the gene *TARDBP* on human chromosome 1. It was first identified as a binding partner of the human immunodeficiency virus TAR DNA element [15]. Although the normal biological functions of TDP-43 are not yet fully understood, it has been implicated in transcriptional repression, exon splicing, and possibly miRNA generation, cell cycle regulation and apoptosis [16].

A pathogenic role for TDP-43 in disease is supported by the identification of mutations in *TARDBP* in familial and sporadic ALS [17], [18], [19], [20], [21], [22], [23]. To date, 29 different missense mutations have been identified in *TARDBP*. In FTLD, where 40% of patients have a family history of neurological disease, other multiple chromosomal loci have been revealed [24]. Here, pathogenic mutations in the tau-encoding *MAPT* gene were the first to be found in familial forms of FTLD-tau [25], [26], [27]. This assisted in the development of both cell culture and animal models to investigate tau-related pathomechanisms [28], [29], [30], [31]. Mutations in *TARDBP* have been reported in a number of patients with FTLD plus ALS [32], [33], [34], [35]. Other more frequent pathogenic mutations have been described in genes including *PGRN* [36], [37], *VCP* [38] and *CHMP2B* [39]. Most of these are associated histopathologically with TDP-43 containing inclusions, however, the mechanistic link between these mutations and TDP-43 deposition is not yet fully understood. Cell culture, as well as yeast and animal models have provided insights into TDP-43 pathology [17], [18], [20], [40], [41], [42]. However, neuronal cell culture models that recapitulate the TDP-43 pathology in FTDL-TDP and ALS are limited.

Proteins that are targeted for degradation are enzymatically tagged with ubiquitin, which promotes disintegration by the proteasome machinery [43]. Pathological protein aggregates are frequently surrounded by proteasomes and lysosomes, however, these fail to fully remove the deposits [44], [45]. In addition, proteasome activity declines with age, and is reduced even more so in neurodegenerative disorders such as Alzheimer's or Parkinson's diseases [43]. However, it remains unknown, if this degradative dysfunction is the cause or the result of neurodegeneration [43], [46]. Reduced proteasome activity results in reduced solubility and fragmentation of TDP-43 in lymphoblastoid cells isolated from ALS patients [18], [20]. Here, we studied the effects of proteasome inhibition induced by benzyloxycarbonyl-l-leucyl-l-leucyl-l-leucinal (MG-132) or lactacystin on TDP-43 in neurons. We show that impairing proteasome activity is sufficient to induce cytoplasmic accumulation and aggregation of TDP-43 in primary hippocampal and cortical neurons, as well as an immortalized motor neuron cell line NSC-34.

## Results

### Proteasome inhibitors, but not other stressors induce cytoplasmic accumulation of TDP-43 in primary neurons

**Figure 1:**
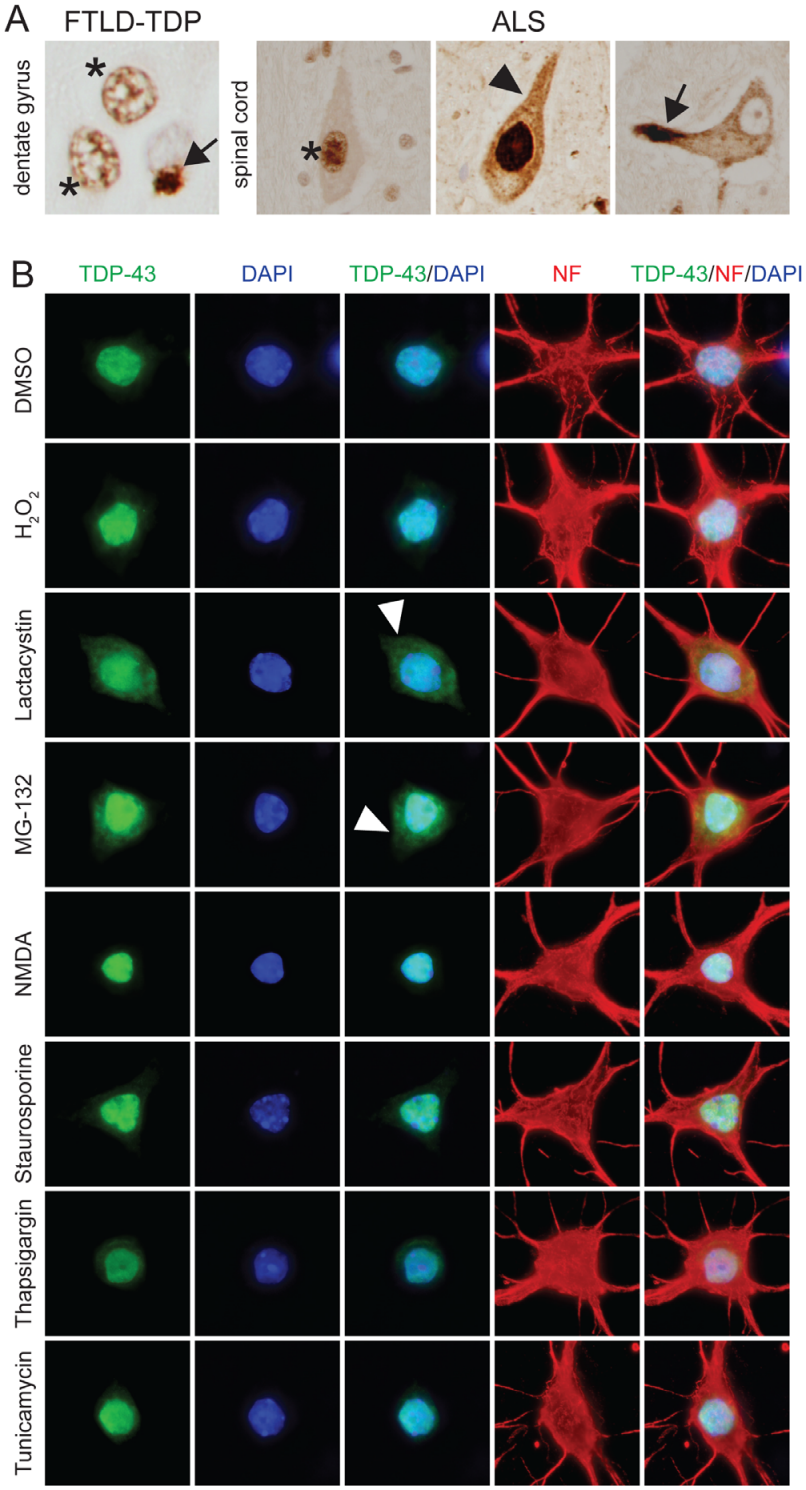
Proteasome inhibition results in cytoplasmic TDP-43 accumulation in cultured neurons. (**A**) Example of immunohistochemical staining for TDP-43 on human frontotemporal lobar degeneration (FTLD) brain and amyotrophic lateral sclerosis (ALS) spinal cord sections. While TDP-43 is normally in the nucleus (asterisk), it accumulates in cytoplasmic aggregates (arrow), while being depleted from the nucleus in affected dentate gyrus neurons in FTLD-TDP. Similarly, TDP-43 that is normally in the nucleus of motor neurons (asterisk), accumulates (arrowhead) and forms cytoplasmic aggregates (arrow) and is depleted from the nucleus in ALS. (**B**) Primary neurons were treated with vehicle (DMSO) or different substances and then stained with antibodies to TDP-43 to assess its subcellular distribution, and neurofilament (NF) for cellular integrity. Doses and treatment times are specified in Materials and methods. TDP-43 localizes primarily to the nucleus upon treatment with H_2_O_2_ (oxidative stress), *N*-Methyl-D-aspartic acid (NMDA, excitotoxicity), staurosporine (caspase activation), thapsigargin or tunicamycine (both endoplasmic reticulum (ER) stress). In contrast, proteasome inhibition with lactacystin or MG-132 treatment induces cytoplasmic accumulation of TDP-43 (arrowheads). DAPI stains nuclei. Representative neurons from three independent experiments are shown.

Redistribution of TDP-43 from the nucleus to the cytoplasm is a characteristic feature found in neurons of brain and spinal cord sections from FTLD-TDP and ALS patients [11] (**Fig. 1A**). The cellular/molecular event(s) that lead to aberrant distribution of TDP-43 in disease remains unknown. It may result from toxic up-stream events that directly affect subcellular localization of TDP-43, it may be associated with cell death, or it may result from the failure of physiological processes that regulate the subcellular distribution of TDP-43. To test this, we treated mature primary murine neurons with a battery of toxins and inhibitors to impair different cellular processes, which also reflect different modes of toxicity implicated in neurodegeneration. The treatments included induction of apoptotic caspase signalling (staurosporine), excitotoxicity (*N*-Methyl-D-aspartic acid (NMDA)), oxidative stress and mitochondrial damage (H_2_O_2_), proteasome inhibition (MG-132, lactacystin) and ER stress (thapsigargin (TG), tunicamycin (TM)). Following treatment, the subcellular distribution of TDP-43 was determined immunocytochemically, complemented by Western blot analysis. Interestingly, only proteasome inhibition with MG-132 or lactacystin resulted in a pronounced change in TDP-43 localization with cytoplasmic accumulation, while TDP-43 remained primarily in nuclei of neurons exposed to staurosporine, NMDA, H_2_O_2_, TG or TM (**Fig. 1B**). All substances caused a similar degree of cell death at the chosen concentrations, but had profoundly different effects on the neurons. Accordingly, Western blot analysis revealed similar phosphorylation of H2AX, a non-specific marker of cell death (**Fig. S1A**), while only MG-132 and lactacystin induced heat shock protein 70 (Hsp70) expression and widespread ubiquitination (**Fig. S1B**).

### Proteasome inhibition increases cytoplasmic TDP-43 in primary neurons

**Figure 2:**
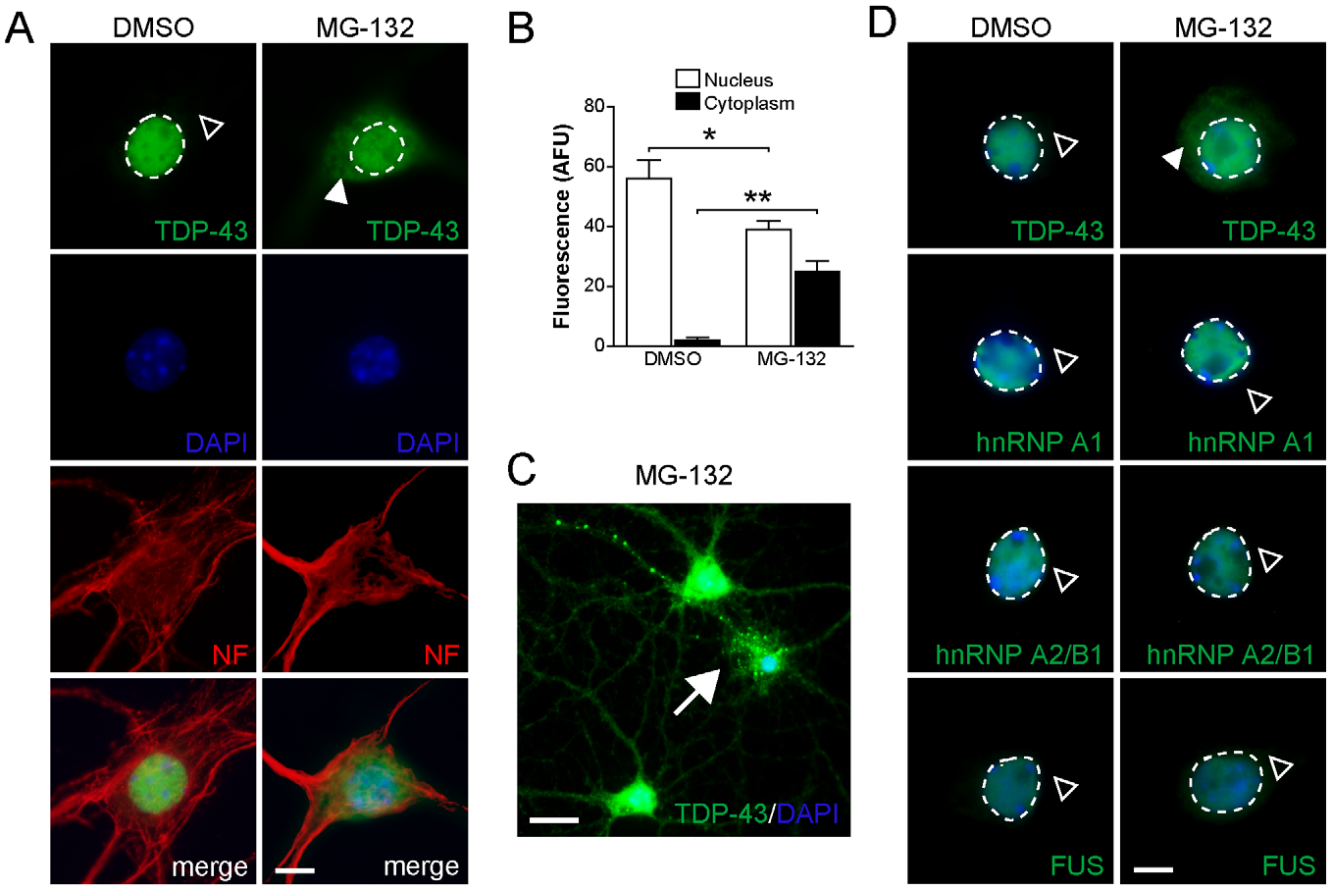
Proteasome inhibition results in cytoplasmic accumulation of TDP-43, but not hnRNP A1, hnRNP A2/B1 or FUS in primary hippocampal neurons. (**A**) TDP-43 expression is restricted to the nucleus (broken line) in vehicle (DMSO) treated primary hippocampal neurons, while being absent from the cytoplasm (open arrowhead). Treatment with 5 µM MG-132 for 12 hours, however, results in cytoplasmic accumulation of TDP-43 (arrowhead), together with reduced nuclear staining. The cellular integrity appears similar in vehicle and MG-132 treated cells, as shown by staining for cytoskeletal neurofilament (NF). Cell nuclei are counterstained with DAPI. Scale bar, 10 µm. (**B**) Quantification of nuclear and cytoplasmic TDP-43 fluorescence staining of vehicle and MG-132 treated primary hippocampal neurons. MG-132 treatment reduces nuclear TDP-43 (Student's *t* test P<0.05; * indicates P<0.01), while cytoplasmic TDP-43 increases markedly (** indicates P<0.001). (**C**) In a subset of neurons, MG-132 induced accumulation of TDP-43 in the cytoplasm is accompanied by focal intensive staining in soma and processes, suggestive of aggregate formation (arrow). Scale bar, 25 µm. (**D**) While TDP-43 accumulates in the cytoplasm upon MG-132 treatment, the nuclear localization of neither hnRNP A1, hnRNP A2/B1 nor FUS is affected (open arrowhead) compared to vehicle (DMSO) treated neurons. Nuclei are stained with DAPI. Scale bar, 10 µm.

Impaired proteasome function has been implicated in neurodegenerative disorders including FTLD-U and ALS, resulting in decreased clearance of mislocalized and misfolded proteins [43]. Given that only proteasome inhibition induced cytoplasmic accumulation of TDP-43 in primary neurons under our experimental conditions (**Fig. 1B**), we analysed these changes in more detail. Therefore, we treated primary hippocampal and cortical neurons, as well as the motor neuron cell line NSC-34 with the 26S proteasome inhibitor, MG-132, and assessed TDP-43 re-distribution, accumulation and aggregation. First, mature hippocampal primary neurons at 20 days *in vitro* (DIV) were treated with 5 µM MG-132 for 24 to 60 hours and analyzed by immunocytochemistry. While TDP-43 localized strictly to the nucleus in vehicle-treated controls, as observed by overlay with DAPI staining, proteasome inhibition resulted in staining of TDP-43 in the cytoplasm together with a reduction in nuclear TDP-43 staining (**Fig. 2A and B**). In a subset of cells with accumulation of TDP-43 in the cytoplasm following proteasome inhibition, we observed focal accumulations that stained intensely with antibodies to TDP-43, suggestive of aggregate formation (**Fig. 2C**). Proteasome inhibition may also induce re-localization of other nuclear proteins besides TDP-43. Therefore, we stained for the closely related nuclear factors hnRNP A1, hnRNP A2/B1 and FUS. In contrast to TDP-43, hnRNP A1, hnRNP A2/B1 and FUS all remained in the nucleus of MG-132 treated cells (**Fig. 2D**). Hence, proteasome inhibition did not affect the localization of nuclear proteins in general.

**Figure 3:**
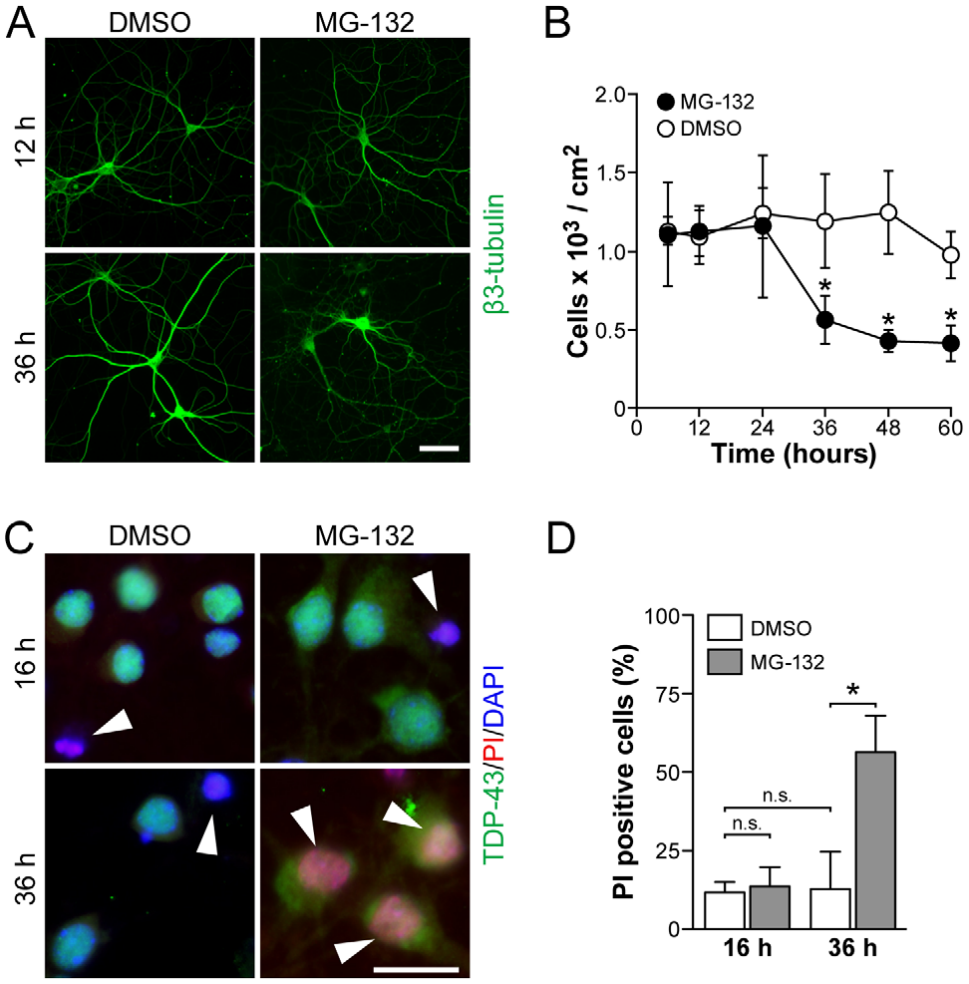
Cell death occurrs after TDP-43 alterations in MG-132 treated primary neurons. (**A**) Staining for neuronal β3-tubulin is similar in vehicle (DMSO) and MG-132-treated neurons after 12 hours, whereas after 36 hours of treatment the staining reveals fragmentation of tubulin in MG-132 treated cells, indicating cell death. Scale bar, 50 µM. Representative pictures from at least three experiments are shown. (**B**) Number of viable attached primary neurons are similar for up to 24 hours of vehicle and MG-132 treatment, but decline significantly thereafter upon MG-132 exposure (* indicates P<0.05; n = 4). (**C**) Immunocytochemical staining with antibodies to TDP-43 combined with propidium iodine (PI) and nuclear counterstaining with DAPI. Cell death is indicated by PI uptake and nuclear staining (arrowheads). Note the cytoplasmic accumulation of TDP-43 upon MG-132 treatment (right panels), and the increased numbers of PI positive neurons after 36 hours of MG-132 exposure (bottom right panel). Scale bar, 25 µM. (**D**) Numbers of PI positive neurons are similar after 16 hours of vehicle and MG-132, and 36 h of vehicle treatment, but significantly increased after 36 hours of MG-132 exposure (Student's t test P<0.05; n.s., not significant; * indicates P<0.05).

While 5 µM MG-132 treatment already resulted in the appearance of cytoplasmic TDP-43 after 12 hours, first signs of degeneration only occurred after 36 hours, as determined by fragmented neuronal β3-tubulin staining in processes (**Fig. 3A**). Counting of neurons revealed that 5 µM MG-132 significantly reduced numbers of viable attached cells only after 36 hours (**Fig. 3B**). Furthermore, we determined the toxicity of MG-132-mediated proteasome inhibition by propidium iodine (PI) uptake, a measure of cell death frequently used in neurons [47], [48]. While both vehicle and MG-132 treatment showed similar numbers of PI-positive cells after 16 hours, this was significantly increased with MG-132, but not vehicle, treatment after 36 hours, suggesting late but not early toxicity (**Fig. 3C and D**). Taken together, treatment of cultured primary neurons with the proteasome inhibitor MG-132 resulted in early cytoplasmic accumulation of TDP-43 followed by late cell death.

### Proteasome inhibition results in re-distribution of TDP-43 in primary cortical neurons

**Figure 4:**
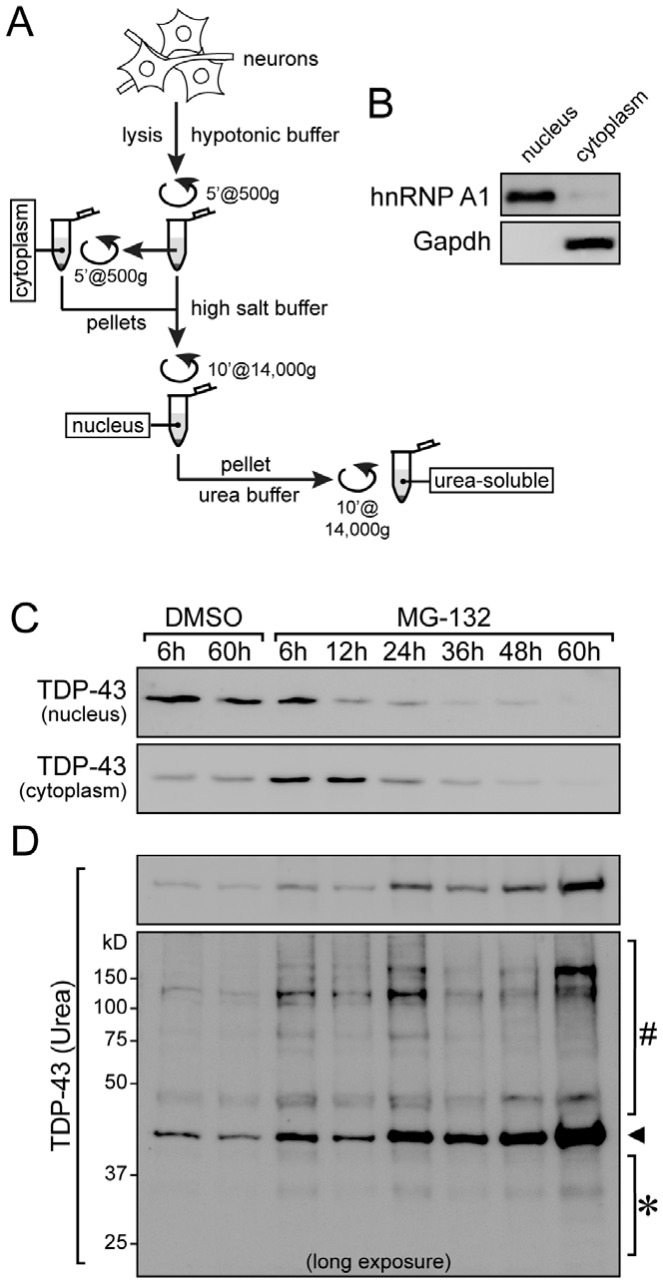
Primary cortical neurons show cytoplasmic accumulation and eventually insolubility of TDP-43 in response to proteasome inhibition. (**A**) Scheme of subcellular extraction into cytoplasmic and nuclear fractions, with subsequent reconstitution of insoluble proteins in urea buffer. For details see *Methods and materials*. (**B**) Western blotting of representative fractionation with nuclear marker hnRNP A1 and cytoplasmic marker Gapdh. (**C**) Subcellular fractionation of vehicle (DMSO) and MG-132-treated primary cortical neurons over 60 hours. TDP-43 is primarily in the nuclear fraction after 6 to 60 hours of vehicle treatment. In contrast, nuclear TDP-43 decreased progressively, and increased transiently in the cytoplasmic fraction upon MG-132 treatment. (**D**) Insoluble pellets from the cytoplasmic fraction were extracted with a urea buffer. This shows increasing amounts of insoluble TDP-43 (arrowhead) during MG-132, but not vehicle treatment. Long exposure of the Western blot reveals the presence of both low molecular weight fragments (*) and high molecular weight aggregates (#) of TDP-43 upon MG-132 treatment. Representative blots of three experiments are shown.

Next, we addressed the subcellular distribution of TDP-43 in cortical neurons following MG-132 treatment. Nuclei of MG-132 and vehicle treated primary cortical neurons were separated from cytoplasmic fractions at different time points by sequential extraction [49] (**Fig. 4A and B**). Consistent with its physiological localization, TDP-43 was predominantly found in the nuclear fraction of vehicle-treated control cells at all time points (**Fig. 4C** **, top**). Similarly, TDP-43 was abundant in the nuclear fraction after 6 hours of incubation with MG-132. However, levels of nuclear TDP-43 progressively decreased after 12 hours of treatment with MG-132, and were undetectable after 60 hours (**Fig. 4C** **, top**). In parallel to the nuclear decrease, TDP-43 was increasingly recovered in cytoplasmic fractions until 12 hours of treatment with MG-132. Interestingly, TDP-43 then also progressively decreased in the cytoplasmic fraction after 24 and 36 hours, and was hardly detectable at 60 hours (**Fig. 4C** **, bottom**). Since there was no obvious difference in the intensity of cytoplasmic TDP-43 staining in MG-132 treated neurons between 16 and 36 hours or even later (**Fig. 2D**), we hypothesized that the late decrease in both nuclear and in particular cytoplasmic TDP-43 revealed by the sequential extraction (**Fig. 4C**) may reflect increasing insolubility of TDP-43, which would therefore not be extracted by the buffer used to recover cytoplasmic proteins. Therefore, we resuspended pellets from the previous fractions in stringent urea buffer (**Fig. 4D**). Consistent with an increased insolubility, the amount of TDP-43 in the urea extracts increased with treatment duration. In addition, higher molecular weight aggregated forms of TDP-43 appeared together with fragments, as revealed by long exposure of membranes (**Fig. 4D** **, bottom**). Taken together, MG-132 treatment of cortical neurons resulted in cytoplasmic accumulation of TDP-43, which eventually became insoluble.

### Increased TDP-43 insolubility, phosphorylation and ubiquitination upon proteasome inhibition in primary neurons

**Figure 5:**
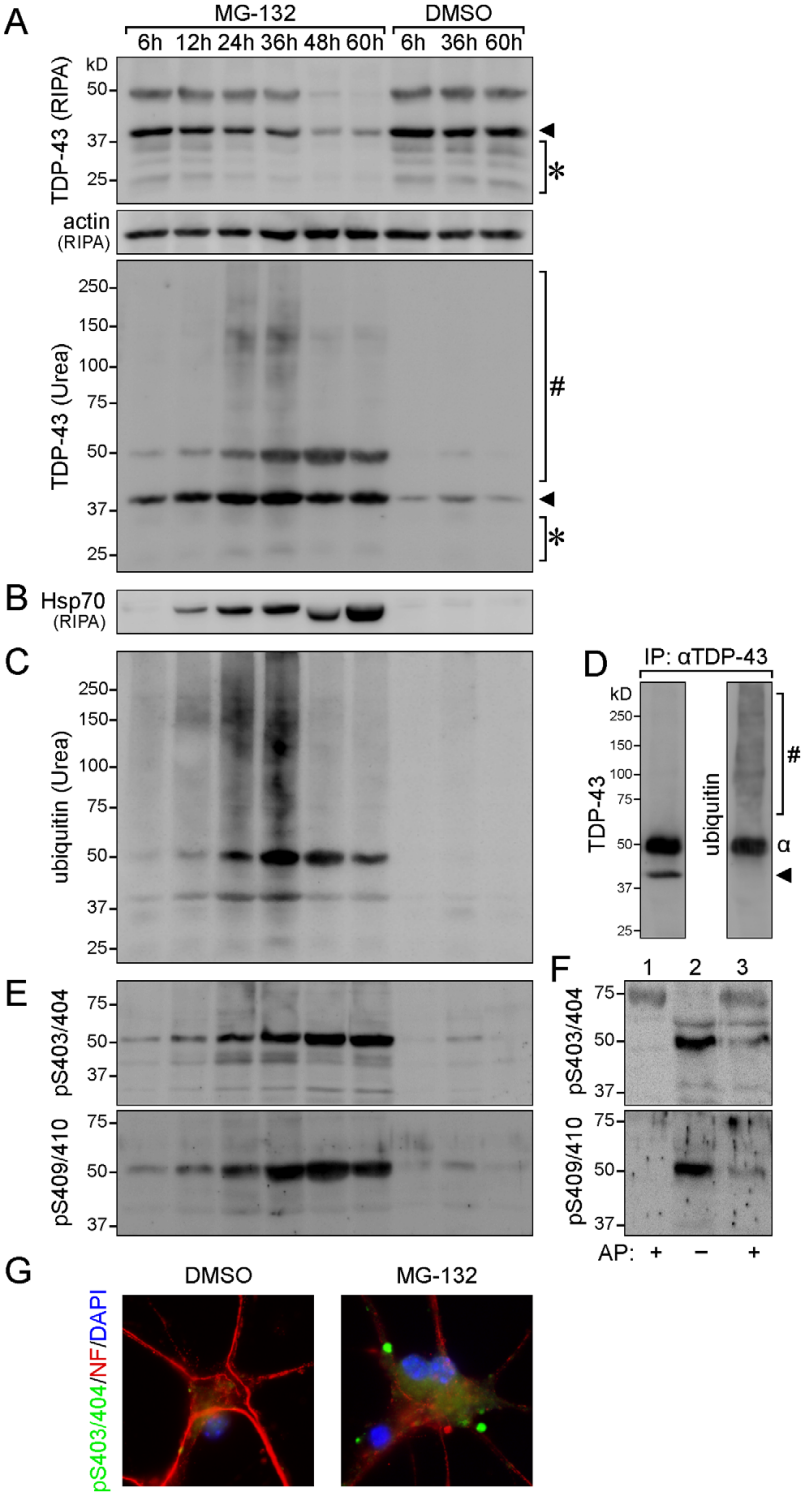
Progressive insolubility of TDP-43 upon proteasome inhibition in primary cortical neurons. (**A**) Proteins were extracted from MG-132 and vehicle (DMSO) treated primary cortical neurons, with RIPA buffer followed by urea extraction of the insoluble pellets. TDP-43 (arrowhead) and its fragments (*) progressively disappear from the RIPA-soluble fractions over 60 hours, whereas no change is observed in vehicle treated controls. Note the presence of soluble TDP-43 fragments in controls. In parallel, TDP-43 (arrowhead) increased in the insoluble (Urea) fractions of MG-132 treated neurons together with fragments (*) and high molecular weight aggregates (#). An additional TDP-43 reactive band of approximately 50kD appears only in primary cortical neurons. (**B**) Consistent with proteasome inhibition by MG-132, levels of heat-shock protein 70 (Hsp70) increase with time. (**C**) Similarly, the amount of insoluble highly ubiquitinated proteins increase progressively upon MG-132 treatment. (**D**) Immunoprecipitation (IP) with an antibody to TDP-43 and subsequent detection with an antibody to ubiquitin shows the high degree of ubiquitination of the high molecular weight TDP-43 reactive species (#). Note the 50kD Fc-antibody band (α). (**E**) Antibodies specific to phosphorylation sites on TDP-43 (pS403/404 and pS409/410) reveal increasing phosphorylation of TDP-43 over time upon MG-132 treatment. Representative Western blots from four experiments are shown. (**F**) Dephosphorylation of urea extracts with alkaline phosphatase (AP) confirms the specificity of the antibodies to S403/4 and S409/10. Lane 1, urea extract of DMSO-treated neurons treated with AP; lane 2, urea fraction of MG-132-treated neurons for 36 hours; lane 3, same extract as lane 2 but treated with AP. (**G**) Similar to the Western blots, immunocytochemical staining with pS403/404 shows cytoplasmic staining in MG-132-treated neurons, different from DMSO-treated cells.

In disease, TDP-43 becomes increasingly insoluble, is aberrantly phosphorylated and shows pronounced ubiquitination [11]. Therefore, we addressed the solubility of TDP-43, as well as its phosphorylation and ubiquitination in primary cortical neurons following treatment with MG-132. The cells were extracted at different time points with RIPA buffer followed by extraction in urea buffer to recover insoluble TDP-43 [50]. In addition to the characteristic 43kD TDP-43 band, we observed an additional band of approximately 50kD that is only present in primary mouse neurons. Treatment with MG-132 resulted in a progressive decrease in soluble TDP-43, with levels hardly detectable after 48 hours (**Fig. 5A** **, top**). No changes in soluble TDP-43 were observed with vehicle treatment. Levels of insoluble 43 kD TDP-43 increased progressively, with highest levels observed after 36 hours (**Fig. 5A** **, bottom**). Western blots of the urea buffer fractions also reveal increased amounts of insoluble high molecular TDP-43 species together with lower molecular weight fragments. Hardly any insoluble TDP-43 was present in the vehicle treated cells. Consistent with impaired proteasome activity, treatment with MG-132 induced expression of Hsp70, which was readily detectable after 12 hours and thereafter (**Fig. 5B**). Proteasome inhibition was accompanied by accumulation of ubiquitinated proteins of decreased solubility (**Fig. 5C**). Immunoprecipitation with an antibody to TDP-43 and subsequent detection of Western blots with an antibody to ubiquitin confirmed the high degree of TDP-43 ubiquitination following proteasome inhibition (**Fig. 5D**). Furthermore, we determined phosphorylation of insoluble TDP-43, with site-specific antibodies. This revealed increased phosphorylation of insoluble TDP-43 at serine (S) 403/404 and S409/410 as the duration of the MG-132 treatment increased (**Fig. 5E**). Dephosphorylation confirmed the specificity of the antibodies used for the respective phospho-epitopes (**Fig. 5F**). Consistent with the Western blots, immunocytochemistry with the phosphorylation site-specific S403/404 antibody showed pronounced staining of cytoplasmic TDP-43 in MG-132-treated cortical neurons, while vehicle-treated control cells showed minimal staining (**Fig. 5G**). Taken together, proteasome inhibition in cortical neurons caused insolubility of TDP-43 in a time-dependent manner, as well as its phosphorylation and ubiquitination.

### Proteasome inhibition induces TDP-43 pathology in NSC-43 cells

Neuronal cell lines have been instrumental in studying both physiological and pathological processes in neurodegeneration [51]. Therefore, we addressed whether treatment with MG-132 would induce TDP-43 insolubility, similar to that observed in primary cultured neurons, in immortalized, murine motor neuron NSC-34 cells [52]. We induced cell cycle arrest with mitomycin C treatment to synchronize all cells prior to treatment with MG-132. Mitomycin C treatment alone had no effect on TDP-43 solubility (data not shown). Proteins were extracted from treated NSC-34 cells based on their solubility, using the same method as described above for primary cortical neurons [49]. Proteasome inhibition leads to a moderate decrease in soluble TDP-43 (**Fig. S2A**). In parallel, it caused a progressive increase in insoluble TDP-43, together with the appearance of high molecular weight aggregates. In comparison, the amount of insoluble TDP-43 in vehicle treated controls was very low. In contrast to the primary neuronal cultures, insoluble 25 and 35kD low-molecular weight fragments were much more abundant in the NSC-34 cells. Similar to the primary neurons, treatment with MG-132 resulted in a progressive increase in Hsp70 expression levels after 12 hours, together with a high degree of protein ubiquitination consistent with proteasome inhibition (**Fig. S2B and C**). Next, we counted the number of viable NSC-34 cells during MG-132 treatment (**Fig. S2D**). Cell numbers were comparable with vehicle until 36 hours of MG-132 treatment, and significantly lower numbers were observed only 48 hours after the commencement MG-132 treatment, suggesting that TDP-43 insolubility precedes cell death.

In addition to the characteristic 43kD band, the insoluble fractions contained increasing amounts of a 45kD TDP-43 reactive product (**Fig. S2A**). Consistent with TDP-43 phosphorylation, dephosphorylation with alkaline phosphatase (AP) of the insoluble fractions from MG-132 treated NSC-34 cells resulted in a molecular weight shift to 43 kD (**Fig. S2E**). Furthermore, immunoprecipitation with a TDP-43 antibody and subsequent Western blotting with an antibody to ubiquitin showed that TDP-43 was highly ubiquitinated following MG-132 treatment of NSC-34 cells (**Fig. S2F**). Taken together, MG-132 treatment of NSC-34 cells results in a progressive increase in the insolubility of TDP-43 associated with phosphorylation and ubiquitination, resulting in an expression pattern very similar to primary neurons and, more importantly, FTLD-TDP and ALS.

### Reducing TDP-43 levels increases proteasome inhibition-induced cell death

**Figure 6:**
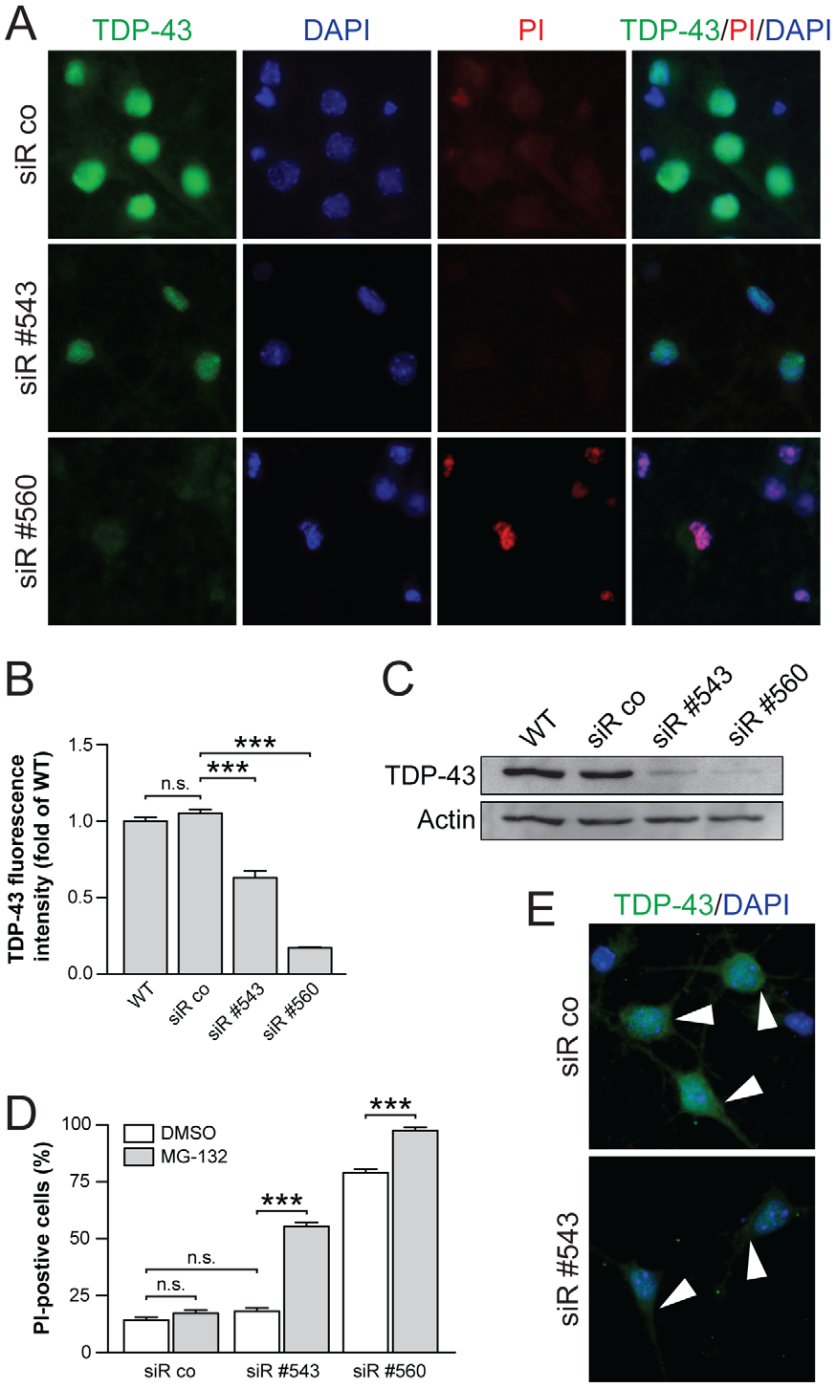
TDP-43 reduction increased vulnerability of neurons. (**A**) Immunocytochemistry of nuclear TDP-43 in neurons expressing control (siR co) or two different siRNAs against TDP-43 (siR #543 and siR #560). SiR #543 and more so siR #560 reduce TDP-43 levels. Propidium iodine (PI) uptake indicates increased cell death in siR #560 neurons. DAPI counterstains nuclei. (**B**) Quantification of TDP-43 fluorescence intensity reveals a 42±5.0% and 88±2.2% reduction of TDP-43 levels with siR #543 and siR #560, respectively, compared to control (siR co) neurons (Student's *t* test P<0.05; *** indicates P<0.0001). Levels of TDP-43 are similar in siR co and wild-type (WT) neurons (n.s., not significant). (**C**) Western blotting confirms reduction of TDP-43 levels with siR #543 and siR #560, compared to siR co and wild-type (WT) neurons. Actin serves as loading control. (**D**) Numbers of PI positive neurons upon treatment with vehicle (DMSO) or MG-132 for 16 hours. Numbers of PI positive cells are similar in DMSO- and MG-132-treated siR co neurons, as well as in DMSO-treated siR #543 cells (Student's *t* test P<0.05; n.s., not significant). MG-132 treatment significantly increases numbers of PI positive siR #543 neurons (P<0.0001). High numbers of PI positive siR #560 neurons (79±1.5%) are also increased by MG-132, with virtually all cells being PI positive (98±1.4%; *** indicates P<0.0001). (**E**) MG-132 induces cytoplasmic accumulation (arrowheads) of TDP-43 in both siR co and siR #543 neurons. Note the faint staining in siR #543 neurons due to reduced TDP-43 levels.

MG-132 treatment of neurons recapitulated major histopathological findings observed in human ALS and FTLD-TDP. The question remains, however, whether this is a secondary effect in cells that eventually die from proteasome inhibition, or if TDP-43 contributes to the cell death following MG-132 treatment. To address this question, we utilized siRNA-mediated knockdown of TDP-43 expression in primary cortical neurons, then treated the cells with MG-132 and determined cell death by PI uptake as described in [48]. To achieve knockdown of TDP-43 in primary neurons, we tested four different siRNA constructs together with a control vector from the MISSION library (Sigma, USA) using lentiviral expression. Two siRNAs (#560 and #543) mediated significant knockdown of TDP-43 in primary neurons with siRNA #560 being more effective than siRNA #543, while control infections did not alter TDP-43 levels, as shown by immunocytochemistry and Western blotting (**Fig. 6A–C**). Accordingly, immunocytochemistry with antibodies to TDP-43 showed reduced staining in virtually all cells following lentiviral infection, compared with controls, with stronger reduction with siRNA #560 than siRNA #543 expression (**Fig. 6A and B**). Similarly, Western blotting showed greater reduction in TDP-43 levels with siRNA #560, than with siRNA #543 (**Fig. 6C**). Control infection did not alter TDP-43 levels. The 42±5.0% reduction of TDP-43 levels caused by siRNA #543 did not affect survival of the neurons, as indicated by similar numbers of PI-positive neurons compared to non- or control-transfected cells (**Fig. 6A and D**). In contrast, the strong reduction of TDP-43 by siRNA #560 reduces viability with increased numbers (79±1.5%) of PI-positive neurons (**Fig. 6A and D**).

Consistent with our previous findings, MG-132 treatment resulted in cytoplasmic re-distribution of TDP-43, with 17±1.4% of control cells being PI positive after 16 hours compared with 14±1.3% PI-positive cells with DMSO treatment (**Fig. 6D**), consistent with survival rates of non-infected MG-132-treated neurons (not shown). However, cells with siRNA #543-mediated knockdown of TDP-43 showed a significantly higher number (55±1.8%) of PI positive cells following MG-132 treatment compared with 18±1.5% of PI-positive cells with DMSO treatment, suggesting increased vulnerability to proteasome inhibition (**Fig. 6D**). SiRNA #560-induced TDP-43 knockdown together with MG-132 treatment caused PI uptake in virtually all cells at 16 hours (**Fig. 6D**). This suggests that the level of TDP-43 reduction correlates with cell death after MG-132 treatment. Re-localization of TDP-43 was not affected by the knockdown, since MG-132 treatment still resulted in cytoplasmic TDP-43, although staining was much less intense due to the reduction in its overall levels (**Fig. 6E**). Taken together, reduced TDP-43 levels increased cell death induced by MG-132 in primary neurons.

## Discussion

In the present study, we demonstrate that inhibition of the proteasome in both primary hippocampal and cortical neurons induces accumulation of the nuclear factor TDP-43 in the cytoplasm and its progressive insolubility. In addition to the 43kD full-length TDP-43 band, the insoluble fractions contained increasing amounts of high molecular weight TDP-43-reactive products, consistent with detergent-stable aggregates. This pattern of aggregates appears similar to that of insoluble extracts obtained from FTLD-TDP and ALS tissues [11]. The inclusions in FTLD-TDP and ALS contain both phosphorylated and ubiquitinated TDP-43 [11], [53], [54]. Similarly, using phosphorylation site-specific antibodies, we show the presence of phosphorylated TDP-43 in insoluble fractions from MG-132 treated cells, while IP confirmed ubiquitination of TDP-43. Interestingly, immunocytochemistry of MG-132-treated neurons shows that phosphorylation of TDP-43 at S403/S404 was significantly increased when TDP-43 localized to the cytoplasm. Similar to primary neurons, proteasome inhibition in immortalized murine motor neurons, NSC-34, resulted in progressive insolubility of TDP-43, with increased phosphorylation, ubiquitination and fragment formation, demonstrating their potential as a cell culture model for an ALS-like TDP-43 pathology. It remains to be determined whether phosphorylation and/or ubiquitination are directly involved in the cytoplasmic accumulation of TDP-43, as it has been suggested for FTLD-TDP and ALS [53], or, alternatively, whether this occurs after TDP-43 accumulates in the cytoplasm. The latter may be supported by the finding that TDP-43 phosphorylation is a late event in a cellular disease model, and therefore not a prerequisite for its aggregation [55].

In addition to the high molecular weight aggregates of TDP-43, proteasome inhibition also induced the formation of lower molecular weight fragments. These fragments were of 25 and 35kD, which is reminiscent of FTLD-U and ALS [11]. It has been suggested that formation of fragments is a critical step in the TDP-43 pathology [20], [56]. Interestingly, fragments of 35kD and, to a lesser degree, 25kD were also revealed in vehicle treated cells, but only became insoluble together with full-length TDP-43 in MG-132 treated cells, which may suggest that fragmentation alone is not sufficient to induce insolubility. A second hit such as proteasome inhibition, appears to be needed to induce insolubility in our system.

Apart from proteasome inhibitors, additional cell stressors have been used to reproduce features of TDP-43 pathology in different cell culture systems. For example, fragmentation of TDP-43 in cell culture has been induced by staurosporine [55], [56] as well as TG [57]. However, mostly immortalized, non-neuronal cell lines and mutant forms of TDP-43 have been used. Given the neuronal pathology, we tested a range of stressors in our primary cell culture system. Consistent with other studies in cell lines, fragmentation of TDP-43 occurred under most conditions, and was very prominent in NMDA-, staurosporine- and H_2_O_2_-treated neurons (data not shown). Interestingly, in our experiments, neither excitotoxicity, caspase activation, oxidative stress nor ER stress induced noticeable accumulation of TDP-43 in the cytoplasm. Therefore, pronounced TDP-43 accumulation in the cytoplasm is, among the tested cell stressors, a unique feature of proteasome inhibition. This possibly reflects that the subcellular distribution of TDP-43 is regulated, at least in part, by the proteasome system. Interestingly, proteasome inhibition is known to change the subcellular localization of a range of proteins in processes like centrosome recruitment and nuclear translocation [58], [59], [60], [61], [62]. A detailed understanding of this process may provide a future understanding of TDP-43 pathology.

TDP-43 has been found to functionally interact with both hnRNP A1 and hnRNP A2/B1 [63]. Although TDP-43 changed its subcellular localization upon MG-132 treatment, that of the closely related nuclear factors hnRNP A1 and hnRNP A2/B1 was not altered, suggesting that the interaction is disrupted during the redistribution of TDP-43. This may have direct implications for neuronal functions, since it has been shown that disruptions to the interactions between TDP-43 and the hnRNP complex interferes with splicing processes [63]. Similar to the hnRNPs, FUS remained within the nucleus in MG-132-treated neurons. These differences in the re-localization of nuclear proteins following proteasome inhibition, may indicate that TDP-43 is shuttled at a much higher rate between the nucleus and cytoplasm, which in turn may cause it to become more prone to cytoplasmic accumulation in cells with a dysfunctional proteasome.

Proteasome dysfunction has been implicated in several neurodegenerative disorders, including ALS [43], [46]. Consistent with our findings, proteasome inhibition resulted in the accumulation of insoluble full-length TDP-43 together with fragments, in lymphoblastoid cells isolated from patients carrying *TARDBP* mutations [18], [20]. Furthermore, studies using a range of immortalized cell lines expressing either wild-type or mutant TDP-43 suggested that proteasome inhibition accelerates TDP-43 accumulation and insolubility [20], [40], [64], [65], [66]. Hence, approaches using proteasome inhibitors provide valuable insights into the effects of TDP-43 mislocalization and insolubility. Since neurons are the primary cell type affected in FTLD-U and ALS, we tested the effects of proteasome inhibition on endogenous TDP-43 in primary cortical and hippocampal neurons or immortalized motor neurons. Interestingly, we found that proteasome inhibition appears to have much greater effects on TDP-43 localization and solubility in primary neurons compared to the immortalized motor neurons. This may result from neurons being postmitotic, while immortalized cell lines undergo cell division that could compensate for cell damage/death.

A common effect of treatments that lead to TDP-43 pathology in cell culture models (e.g. TDP-43 fragmentation or subcellular re-distribution) is that they also induce cell death, which is sometimes not taken into account. Hence, the question remains as to whether the induction of the TDP-43 pathology is a result of cell death (or pathways that co-induce cell death and TDP-43 pathology), or alternatively, that the TDP-43 pathology contributes to the cell toxicity. Also in our study, we cannot fully exclude that cell death is accompanied by TDP-43 re-localization, however, these changes were observed prior to detection of cellular toxicity. Furthermore, moderately reducing TDP-43 levels in primary neurons via siRNA increased their vulnerability to MG-132 treatment significantly, while only strong reduction of TDP-43 levels by siRNA induced cell death on its own. Knockdown of TDP-43 induces cell death in Neuro-2a cells possibly through Rho family GTPases [67]. However, the exact molecular mechanisms underlying TDP-43 pathology-induced toxicity in neurons remain to be identified. To this end, our data indicates that loss of TDP-43 function contributes to neurodegeneration, and makes neurons more vulnerable to second hits, such as proteasome dysfunction.

In summary, we showed that proteasome inhibition by MG-132 in primary neurons and the NSC-34 motor neuron cell line is sufficient to induce cytoplasmic accumulation, aggregation, fragmentation and insolubility of TDP-43, bearing similarities to the TDP-43 pathology in FTLD-TDP and ALS. Reducing TDP-43 levels exacerbated the toxic effects of MG-132-induced proteasome inhibition in cultured neurons. Therefore, our data support a second hit hypothesis in addition to TDP-43 dysfunction in these diseases. Furthermore, our data suggest that the subcellular distribution of TDP-43 under physiological conditions is, at least in part, regulated by the proteasome system.

## Materials and Methods

### Human samples

Human brain samples were obtained from the Australian Brain Bank Network and the Department of Pathology of the University of Zurich, Switzerland, with approval from the Human Ethics Review Committee of the University of Sydney and the Zurich University Hospital Research Ethics Committee. Written consent from donors or donors next of kin for brain donation was received by each brain bank. Immunohistochemical staining was carried out as previously described [68].

### Cell culture

All animal experiments have been approved by the Animal Ethics Committee of the University of Sydney (approval number K00/1-2010/3/5186). Primary hippocampal and cortical cultures were prepared as previously described [51]. Briefly, hippocampi and cortices from E16.5 mouse embryos were harvested, trypsinized and dissociated in HBSS containing 1mg/ml DNase and trypsin (Sigma) at 37°C for 20 minutes. For immunocytochemistry, 200 000 cortical cells were plated in 10% FBS/DME (Hyclone) in a ring around 2500 hippocampal or cortical cells on 12 mm glass coverslips coated with poly-D-lysine (Sigma). This cortical ring provides neurotrophic support for the hippocampal cells. For Western blotting 2×10^6^ cortical cells were plated on 10 cm culture dishes. 2 hours after cells have attached, the plating medium was exchanged to Neurobasal medium supplemented with B-27 supplement and Glutamax (all Gibco).

The immortalized motor neuron cell line NSC-34 was cultured as previously described [52]. For Western blotting and cell counting, 1×10^6^ NSC-34 cells were plated on 10cm culture dishes and maintained in medium containing high glucose DME (Gibco, Invitrogen, USA) supplemented with 10% FBS (Hyclone, USA), 2 mM L-glutamine and penicillin/streptomycin (Gibco).

### Cell culture treatments

Primary hippocampal and cortical cultures were treated with 5 µM MG-132 (Calbiochem, Merck), 10 µM lactacystin (Calbiochem), 20 µM thapsigargin and 100 µg/mL tunicamycin (both Sigma) after 20 DIV for up to 60 hours. These compounds were dissolved in DMSO to yield stock solutions. For all subsequent treatments, the compounds and DMSO were further diluted in Neurobasal/B-27 medium and added to cultures to the indicated final concentrations. For treatment with 5 µM H_2_O_2_, 1 µM NMDA and 50 nM staurosporine (all Sigma), the compounds were dissolved in either sterile water (H_2_O_2_ and NMDA) or DMSO (staurosporine) to yield stock solutions, and for all subsequent treatments further diluted in Neuralbasal/B-27 medium. Treatments with these particular compounds were added directly to the culture medium for 1 hour, after which the medium was removed, the cultures washed once with culture medium, and then replenished with fresh Neuralbasal/B-27 medium and were then continued to be cultured for the indicated amount of time. For vehicle controls, the according final dilution of DMSO was used.

Prior to treatment with MG-132, the NSC-34 cells were treated with Mitomycin C (Sigma) at a final concentration of 10 ug/mL for 2.5 hours to arrest cell division. As for primary cultures, stock solutions of MG-132 and DMSO were dissolved in culture medium and cells treated at the indicated concentrations. For cell counting, cell were washed once with pre-warmed PBS to remove any unattached cells, and stained with 0.4% trypan blue solution (Sigma).

### Immunocytochemistry

Once the cultures had been treated for the required amount of time, the cells were washed once with pre-warmed PBS and fixed with 4% paraformaldehyde for 15 min at room temperature. Fixed cells were permeabilized with 0.2% Triton X in PBS for 5 minutes and blocked for 1 hour in 3%BSA/2% heat-inactivated goat serum (Sigma) in PBS. Cells were stained overnight at 4°C with primary antibodies to: TDP-43 (1∶400, Proteintech), phosphorylated TDP-43 Ser403/4 (1∶1000, CosmoBio) hnRNP A1 and hnRNP A2/B1 (1∶200, Sigma), neurofilament (1∶200, Abcam), MAP2 (1∶200, Sigma) FUS (1∶200, Proteintech) and β3 tubulin (1∶300, Covance). Nuclei were visualized with DAPI (1∶1000, Molecular Probes). Secondary antibodies (1∶250, Invitrogen) were coupled with the fluorochromes Alexa Fluor 555 (mouse) and 488 (rabbit). All antibodies were diluted in blocking buffer. Dead cells were visualized by uptake of propidium iodide (Sigma) that had been added to the cell culture medium 5 min prior to fixation [48]. Pictures were taken with a BX51 microscope (Olympus). Fluorescence intensity was quantified using the Image J software (NIH). Intact cortical neurons were counted on images collected from random fields and normalized to the total cell number.

### Subcellular fractionation

Subcellular fractionation of primary cortical cells (20 DIV) treated with MG-132 or DMSO was carried out as previously described [49]. Cells were washed once with pre-warmed PBS, harvested and lysed with hypotonic buffer (10 mM HEPES pH 7.9, 10 mM KCl, 0.1 mM EDTA, 0.1 mM EGTA, 1 mM DTT and Roche Complete Protease Inhibitors). After 20 minutes incubation on ice, cells were homogenized 5 times with 0.3 ml Insulin syringes. Homogenates were centrifuged at 500 *g* for 5 minutes to isolate nuclei. The cytoplasmic supernatant was re-centrifuged at 500 g for 5 minutes to remove any contaminating nuclei. The nuclei pellets were resuspended in high salt buffer (20 mM HEPES pH 7.9, 400 mM NaCl, 1 mM EDTA, 1 mM EGTA, 1 mM DTT and protease inhibitors) and incubated on ice for 20 minutes. This homogenate was centrifuged at 14000 rpm for 10 minutes, at 4°C, to obtain the nuclear supernatant. The pellet was resuspended in urea buffer (7 M urea, 2 M thiourea, 4% CHAPS, 30 mM Tris, pH 8.5), sonicated at 30% amplitude and centrifuged at 14000 rpm. The supernatant obtained is the insoluble fraction.

### Protein extraction

Proteins were extracted from both primary cortical neurons and NSC-34 cells as previously described [50]. Cells were washed once with pre-warmed PBS, harvested and then lysed with ice-cold, modified RIPA buffer (with Roche Complete Protease Inhibitors, 20 mM MOPS (pH 7.0), 1% (v/v) TritonX100, 0.25% Na-deoxycholate, 0.1% SDS, 150 mM NaCl, 2 mM EGTA, 5 mM EDTA, 30 mM NaF, 60 mM β-glycerophosphate, 20 mM Na-pyrophosphate, 1 mM Na_3_VO_4_, 5 µM Pepstatin A, 1 mM DTT). The samples were then sonicated at 30% amplitude and centrifuged at 50,000 *g* for 60 min at 4°C. The supernatant was collected as the soluble fraction. The resulting pellets were resuspended in RIPA buffer, re-sonicated and re-centrifuged as above twice more to remove any contaminating proteins. Only supernatants from the first centrifugation were analyzed. The remaining pellets were resuspended in urea buffer, sonicated at 30% amplitude and centrifuged at 50,000 *g* for 60 min at 4°C. The supernatant obtained is the insoluble fraction.

### Western blotting

Protein measurements of all obtained fractions were determined by the Bradford assay. Protein extracts were analyzed by immunoblotting, which was performed as previously described [69], [70]. Between 10–100 µg of proteins were loaded and separated in 8–15% SDS-PAGE gels. Proteins were then electrophoretically transferred onto nitrocellulose membranes (Hybond, GE Healthcare). These membranes were subsequently blocked with 5% bovine serum albumin (BSA) in Tris-buffered saline containing 0.1% Tween 20 (TBS-T) at room temperature for 1 hour, followed by incubation with the primary antibodies diluted in 5% BSA/TBS-T overnight at 4°C while shaking. Antibodies used were to: TDP-43 (1∶1000, Proteintech), Hsp70 (1∶1000, Stressgen), ubiquitin (1∶2500, Dako), GAPDH (1∶5000, Millipore), α-actin (1∶5000, Chemicon) and α-tubulin (1∶5000, Sigma) H2AX and phosphorylated H2AX (both 1∶1000, Millipore), phosphorylated TDP-43 at Ser403/404 and at Ser409/10 (both 1∶1000, CosmoBio) and hnRNP A1 (1∶1000, Sigma). After removal of the primary antibody, the membranes were washed with TBS-T and then incubated with alkaline phosphatase-coupled secondary antibodies (mouse or rabbit IgG, Sigma) in 1% BSA/TBS-T (1∶15 000) for 30 min at room temperature. Protein bands were visualised with Immobilon Chemiluminescent Alkaline Phosphatase substrate (Millipore) and detected in a VersaDoc Model 4000 CCD camera System (BioRad). For normalisation, the membranes were stripped by washing with dH_2_O for 5 min, followed by 0.2 M NaOH for 10 min and dH_2_O for 5 min before being reprobed for GAPDH, α-actin and α-tubulin. Quantification of bands was performed using BioRad Quantity One 1-D analysis software v4.6 (BioRad).

### Immunoprecipitation

IPs were carried out as previously described [71] using a TDP-43-specific antibody produced in rabbit (Proteintech) for coprecipitation with ubiquitin in IP buffer (50 mM Tris-HCl (pH 8.0), 150 mM NaCl, 1% Nonidet P-40 substitute (all Sigma), and complete EDTA-free protease inhibitor mixture (1 tablet in 40 mL; Roche). Antibodies were pulled down with magnetic Dynabeads Protein G (Invitrogen) and washed four times with IP buffer before eluting with sample buffer for SDS-PAGE.

### AP treatment

Protein samples were incubated with calf intestine AP (Takara, final concentration of 0.28 units/µL) in the corresponding AP buffer (50 mM Tris-HCl (pH 9.0), 1 mM MgCl, and Complete Protease Inhibitor (Roche)) at 37°C for 2 hours while shaking. Once complete, sample buffer was added and the samples analysed by Western blotting.

### Lentiviral production

For lentiviral production, 293T human embryonic kidney cells were used to generate lentiviral packaging, whilst SH-SY5Y cells were used for tittering of viral stocks, as previously described [72]. After 7 DIV, the medium was removed from the primary cultures and replaced with lentiviruses diluted in Neuralbasal/B-27. After 3 days of incubation with the lentivirus the medium was replaced with fresh Neuralbasal/B-27, and after another 5 days the cells were then treated with either 5 µM MG-132 or DMSO for the appropriate amount of time.

### Statistics

Statistical analysis was performed with the Prism 5.0 software (Graph Pad, USA). All values are given as mean ± SEM.

## Supporting Information

Figure S1 Effects of different treatments on primary neurons. (**A**) Western blot analysis reveals similar levels of H2AX upon all treatments: vehicle (DMSO), NMDA (1 µM), H_2_O_2_ (5 µM), staurosporine (50 nM), MG-132 (5 µM), lactacystin (10 µM), thapsigargin (TG; 20 µM) and tunicamycin (TM; 100 µg/mL). While H2AX is not phosphorylated in vehicle-treated neurons, all other treatments caused a similar degree of phosphorylation of H2AX, a non-specific marker of cell death, at chosen doses. (**B**) Only MG-132 and lactacystin induce expression of Hsp 70 and high levels of ubiquitination, indicating proteasome dysfunction.
(TIF)

Figure S2 Progressive insolubility of TDP-43 upon proteasome inhibition in the immortalized motor neuron cell line, NSC-34. (**A**) RIPA and subsequent urea extraction of proteins from MG-132 (5µM) and vehicle (DMSO) treated NSC-34 cells. TDP-43 (arrowhead) and its fragments (*) reduce in the RIPA-soluble fractions over 48 hours, whereas TDP-43 is unchanged in vehicle treated controls. Note the presence of soluble TDP-43 fragments in controls. In parallel, TDP-43 increased in the insoluble (Urea) fractions of MG-132 treated neurons together with fragments (*), a distinct 45kD species (§) and high molecular weight aggregates (#). (**B**) Consistent with proteasome inhibition by MG-132, levels of the heat-shock protein 70 (Hsp70) increases with time. (**C**) Similarly, the amount of insoluble highly ubiquitinated proteins increased progressively upon MG-132 treatment. Representative Western blots from three experiments are shown. (**D**) NSC-34 cell viability during proteasome inhibition. The number of attached viable NSC-34 cells was similar upon vehicle (DMSO) and MG-132 treatment up to 36 hours, while a significant decrease became only obvious after 48 hours (*P<0.01; n = 6). (**E**) Phosphorylation of insoluble TDP-43 from MG-132-treated NSC-34 cells as shown by molecular weight shift upon dephosphorylation with alkaline phosphatase (AP). Accordingly, TDP-43-reactive bands of approximately 45kD (§) and the high molecular weight smear (#) collapsed to 43kD. (**F**) Immunoprecipitation (IP) with an antibody to TDP-43 and subsequent detection with an antibody to ubiquitin shows the high degree of ubiquitination of the high molecular weight TDP-43 reactive species (#). Note the 50kD Fc-antibody band (α).
(TIF)
