# Supplementary figures and images for: Cytoplasmic Accumulation and Aggregation of TDP-43 upon Proteasome Inhibition in Cultured Neurons
Source: PLoS One. 2011 Jul 29;6(7):e22850. doi: 10.1371/journal.pone.0022850 (PMC3146516; doi:10.1371/journal.pone.0022850)

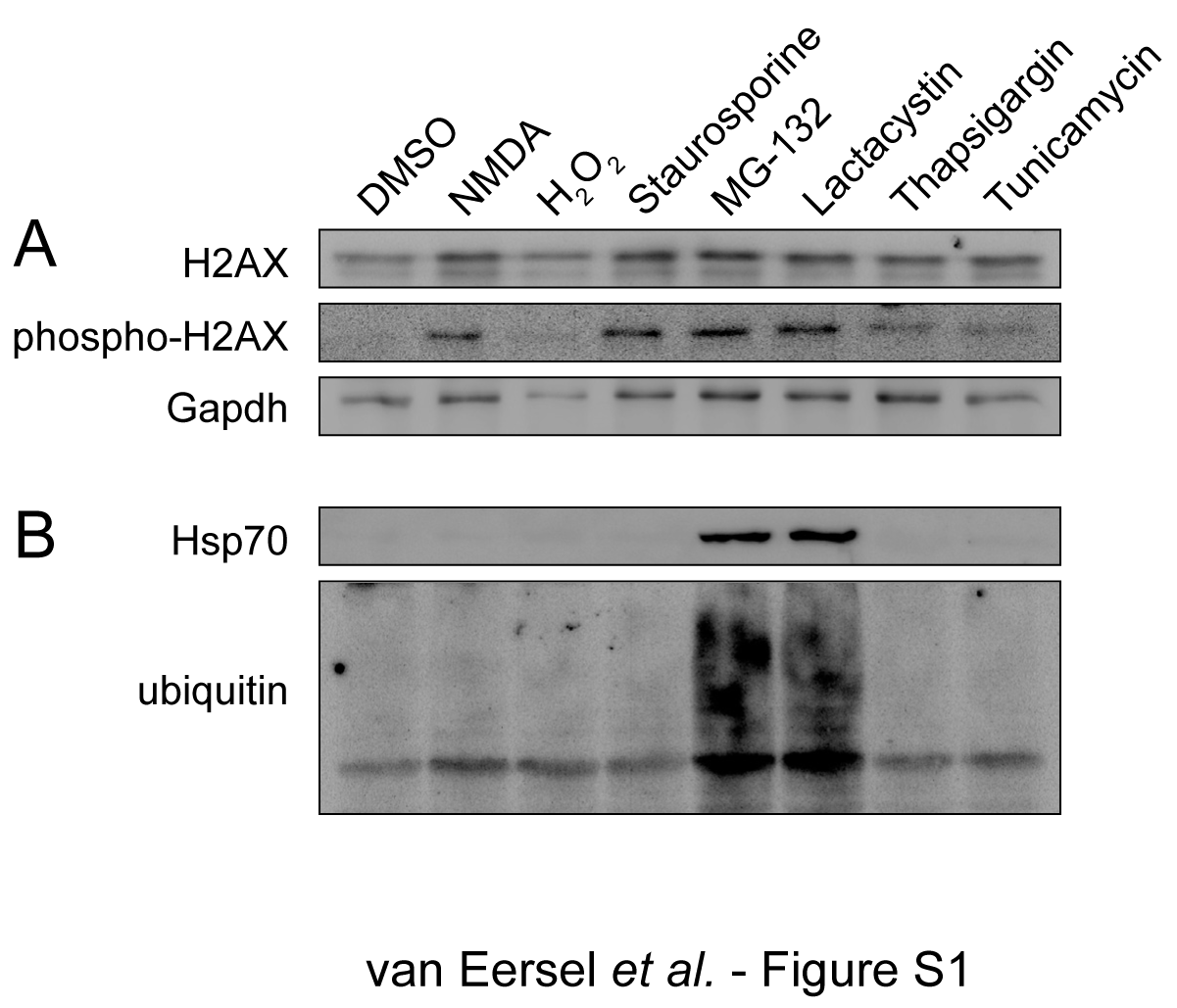

Supplement: Figure S1 — Effects of different treatments on primary neurons. (A) Western blot analysis reveals similar levels of H2AX upon all treatments: vehicle (DMSO), NMDA (1 µM), H2O2 (5 µM), staurosporine (50 nM), MG-132 (5 µM), lactacystin (10 µM), thapsigargin (TG; 20 µM) and tunicamycin (TM; 100 µg/mL). While H2AX is not phosphorylated in vehicle-treated neurons, all other treatments caused a similar degree of phosphorylation of H2AX, a non-specific marker of cell death, at chosen doses. (B) Only MG-132 and lactacystin induce expression of Hsp 70 and high levels of ubiquitination, indicating proteasome dysfunction. (TIF) [file pone.0022850.s001.tif]

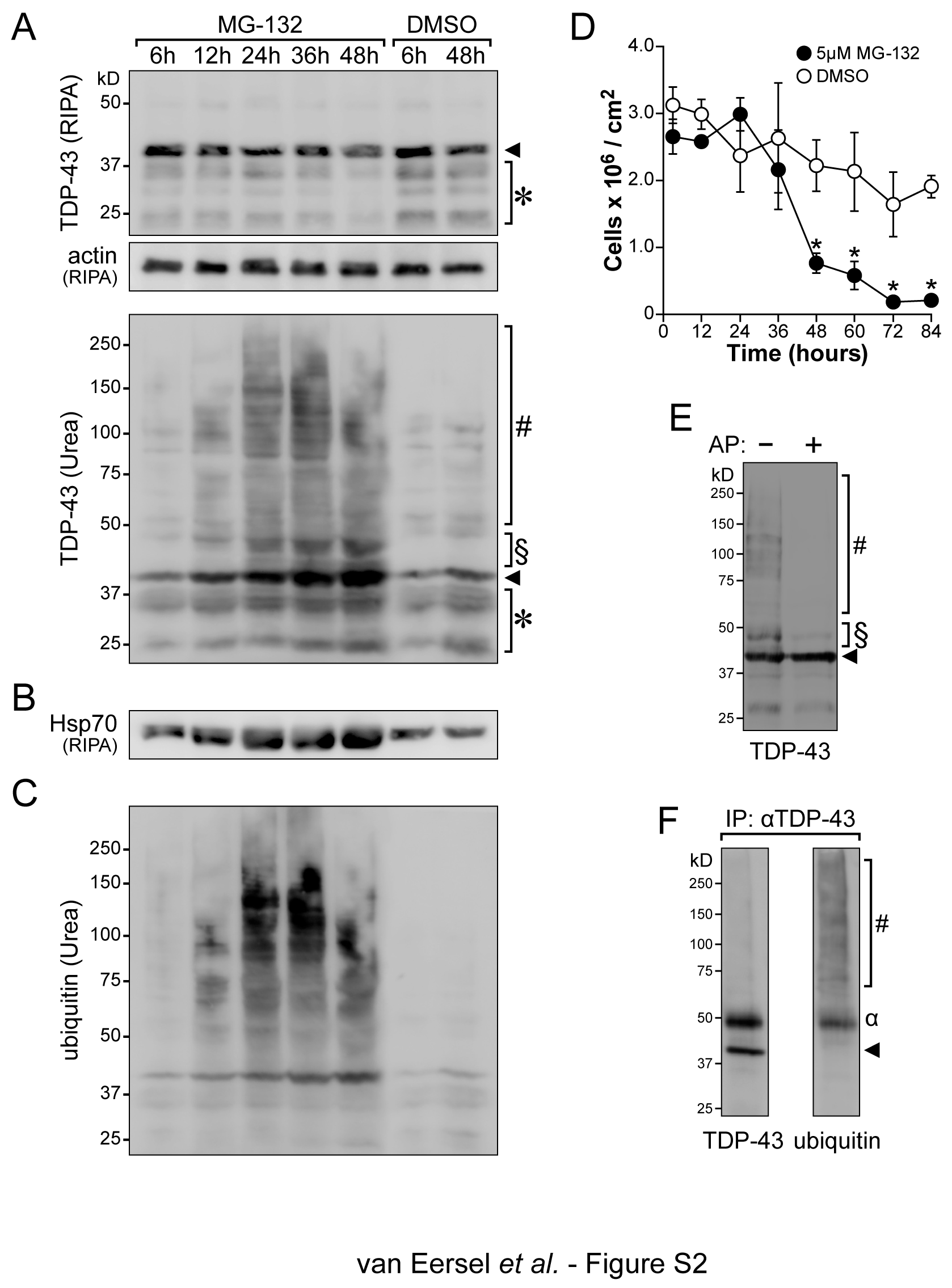

Supplement: Figure S2 — Progressive insolubility of TDP-43 upon proteasome inhibition in the immortalized motor neuron cell line, NSC-34. (A) RIPA and subsequent urea extraction of proteins from MG-132 (5µM) and vehicle (DMSO) treated NSC-34 cells. TDP-43 (arrowhead) and its fragments (*) reduce in the RIPA-soluble fractions over 48 hours, whereas TDP-43 is unchanged in vehicle treated controls. Note the presence of soluble TDP-43 fragments in controls. In parallel, TDP-43 increased in the insoluble (Urea) fractions of MG-132 treated neurons together with fragments (*), a distinct 45kD species (§) and high molecular weight aggregates (#). (B) Consistent with proteasome inhibition by MG-132, levels of the heat-shock protein 70 (Hsp70) increases with time. (C) Similarly, the amount of insoluble highly ubiquitinated proteins increased progressively upon MG-132 treatment. Representative Western blots from three experiments are shown. (D) NSC-34 cell viability during proteasome inhibition. The number of attached viable NSC-34 cells was similar upon vehicle (DMSO) and MG-132 treatment up to 36 hours, while a significant decrease became only obvious after 48 hours (*P<0.01; n = 6). (E) Phosphorylation of insoluble TDP-43 from MG-132-treated NSC-34 cells as shown by molecular weight shift upon dephosphorylation with alkaline phosphatase (AP). Accordingly, TDP-43-reactive bands of approximately 45kD (§) and the high molecular weight smear (#) collapsed to 43kD. (F) Immunoprecipitation (IP) with an antibody to TDP-43 and subsequent detection with an antibody to ubiquitin shows the high degree of ubiquitination of the high molecular weight TDP-43 reactive species (#). Note the 50kD Fc-antibody band (α). (TIF) [file pone.0022850.s002.tif]
